# Experimental Food Restriction Reveals Individual Differences in Corticosterone Reaction Norms with No Oxidative Costs

**DOI:** 10.1371/journal.pone.0110564

**Published:** 2014-11-11

**Authors:** Ádám Z. Lendvai, Jenny Q. Ouyang, Laura A. Schoenle, Vincent Fasanello, Mark F. Haussmann, Frances Bonier, Ignacio T. Moore

**Affiliations:** 1 Department of Biological Sciences, Virginia Tech, Blacksburg, Virginia, United States of America; 2 The Netherlands Institute of Ecology (NIOO-KNAW), Wageningen, The Netherlands; 3 Department of Biology, Bucknell University, Lewisburg, Pennsylvania, United States of America; 4 Department of Biology, Queen's University, Kingston, Ontario, Canada; CNRS, France

## Abstract

Highly plastic endocrine traits are thought to play a central role in allowing organisms to respond rapidly to environmental change. Yet, not all individuals display the same degree of plasticity in these traits, and the costs of this individual variation in plasticity are unknown. We studied individual differences in corticosterone levels under varying conditions to test whether there are consistent individual differences in (1) baseline corticosterone levels; (2) plasticity in the hormonal response to an ecologically relevant stressor (food restriction); and (3) whether individual differences in plasticity are related to fitness costs, as estimated by oxidative stress levels. We took 25 wild-caught house sparrows into captivity and assigned them to repeated food restricted and control treatments (60% and 110% of their daily food intake), such that each individual experienced both food restricted and control diets twice. We found significant individual variation in baseline corticosterone levels and stress responsiveness, even after controlling for changes in body mass. However, these individual differences in hormonal responsiveness were not related to measures of oxidative stress. These results have implications for how corticosterone levels may evolve in natural populations and raise questions about what we can conclude from phenotypic correlations between hormone levels and fitness measures.

## Introduction

**Figure 1:**
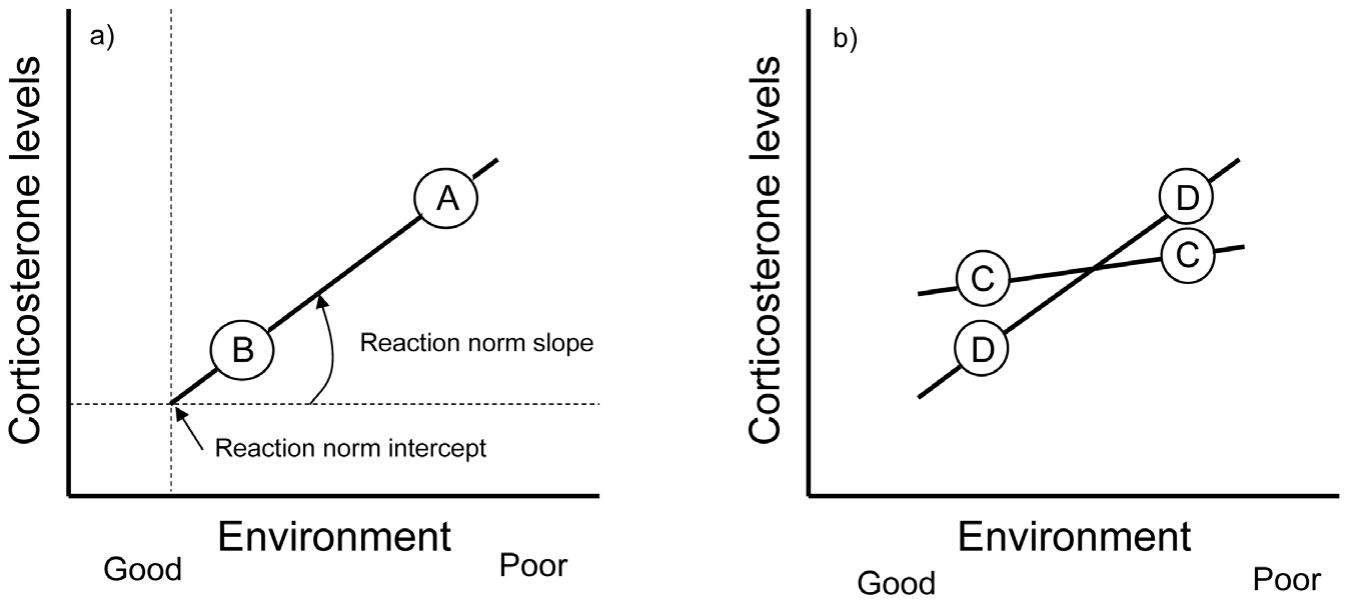
The concept of reaction norms. Reaction norms are the phenotypic expression of a single genotype across a range of environments. Reaction norms can be characterized by the intercept (e.g. initial hormone levels) and the slope of the line (e.g. stress responsiveness). Panel (A) depicts a hypothetical scenario, where there is only one reaction norm in the population (e.g. due to the lack of genetic variance or due to a strong selection for an optimal reaction norm). In this scenario, selection cannot act on hormone levels, because there is no variance between genotypes (individuals A and B have the same slope and intercept), i.e., all individuals will have identical hormone levels under the same environmental circumstances. However, without knowing the reaction norm, differences between the individuals (denoted by A and B) may be mistakenly attributed to results of natural selection on the hormone levels (if fitness is higher in the good environment than in the poor one). In panel (B), individuals differ in their reaction norms (both in the intercept and the slope). Different individuals (C and D) are sampled twice, once in a good environmental condition and once in a bad environmental condition. In this case, the between- and within-individual variation in hormone levels can be separated, and the reaction norm can be defined. If fitness related traits are also measured, then we can ask whether selection acts on the different reaction norms. In this special case, the reaction norms cross, making an individual by environment (I×E) interaction that has further consequences on how natural selection can act on the reaction norms in such a situation. See the text and references therein for further details.

A central goal in evolutionary ecology is to characterize patterns of selection on the optimal phenotype for a given environment. Currently, there is a growing interest in the causes and consequences of between- and within-individual variation in labile traits, including behavior and physiology [1]–[3]. Hormones are labile phenotypic traits, and therefore show a reaction norm (i.e., variable phenotypic expression of a single genotype depending on the environment) [4] (Fig. 1). A number of studies have identified strong phenotypic correlations between plasma hormone concentrations and fitness measures and conclude that these correlations are evidence of natural selection [5]–[8]. However, these trait-fitness correlations and selection coefficients may be confounded or overestimated because of individual plasticity [9]. Specifically, the observed between-individual and fitness-trait correlations could be an artifact of biased sampling [10] or the result of unmeasured traits that are tightly correlated with both the focal trait and fitness, including developmental history [11] and environmental conditions [12]. To illustrate this point, consider the case of hormone levels, which are often adjusted as a response to changes in the environment. If natural selection strongly favors a single optimal hormonal response to the environment such that all individuals in the population have the same reaction norm, then any phenotypic correlation between fitness and hormone levels must be due to the influence of the environment on both the optimal hormone levels and fitness, a scenario that is likely if individuals in a sample are confronted with heterogeneous environmental challenges (Fig. 1). Without knowing individual reaction norms, researchers may be tempted to interpret such phenotypic correlations as evidence of natural selection [13]. This conclusion may be premature or unsubstantiated [14], [15]; therefore, we need to measure the adaptive value of among individual variation in reaction norms of labile traits under different environmental conditions to understand how selection shapes plasticity of these traits.

Glucocorticoids, a conserved family of vertebrate steroid hormones, are secreted in response to changes in environmental and social conditions and prepare the organism to cope with challenges. Thus, these hormones vary along a reaction norm, and circulating concentrations depend on both extrinsic and intrinsic factors, including a wide array of environmental factors 16–18, the development of the Hypothalamic-Pituitary-Adrenal axis (HPA) [19], [20], the value of reproduction [21], [22], and the age of the individual [23], [24]. Although this hormonal response is plastic, there is individual consistency in the stress responsiveness of captive and wild animals 25–30 and artificial selection experiments and a recent cross-fostering experiment have provided evidence for heritability in some of the individual variation in responsiveness [28], [31]. Therefore, in natural populations, endocrine responsiveness itself might be a trait that has been, or is being, shaped by selection. For example, a recent phylogenetic comparative analysis of more than a hundred bird species found that baseline and stress-induced corticosterone (the major glucocorticoid in birds) levels evolved together and resulted in very diverse reaction norms in different species [32]. Similarly, natural selection can act on reaction norms within a single species to optimize the physiological states of individuals across multiple environments [4].

Although glucocorticoid levels change in response to a variety of environmental factors, most of the studies on stress physiology in wild birds have used the capture-handling stress protocol [33], which provides valuable information, but represents an unlikely event for individuals living in the wild [34]. Food shortage, on the other hand, may be a more common and prolonged stressor for most wild birds. In fact, an association between limited food availability and increased corticosterone levels has been shown in wild birds [35], [36]. The close and recurrent association between environmental conditions and corticosterone suggests that this hormonal responsiveness is adaptive [37].

To measure the costs of different corticosterone reaction norms, we can use oxidative stress parameters as surrogate measures for cellular integrity. Managing oxidative stress appears to be an important mediator of life history trade-offs, with wide reaching consequences for individual performance [38]–[43]. For example, the accumulation of oxidative damage over time is thought to be an important contributor to the aging process [38], [44]. Moreover, recent connections established between glucocorticoids, oxidative stress, and aging [20], [45], [46] suggest that some of the effects of oxidative stress on survival may be mediated in part through glucocorticoids [47]–[49].

In this study, we experimentally varied food availability in captive house sparrows (*Passer domesticus*) to test the hypothesis that there is adaptive among-individual variation in the corticosterone reaction norm. We addressed this hypothesis through three main questions. First, we tested whether there are consistent between-individual differences in corticosterone reaction norms. Second, we tested whether there is a covariance between the intercept and slope of an individual corticosterone reaction norm. Chronically elevated baseline corticosterone is often associated with decreased stress responsiveness [50], therefore, the elevation and the slope of the reaction norm may not evolve independently. Finally, we asked whether hormonal responsiveness was related to levels of oxidative stress. Under the hypothesis that hormonal plasticity is adaptive, we expected that natural selection would strongly select for a plastic phenotype; we predicted that the most responsive individuals would have the lowest signs of oxidative stress.

## Methods

### Ethics Statement

All procedures used in this study were approved by the Virginia Tech Institutional Animal Care and Use Committee and free-living bird capture was approved by Virginia's Department of Game and Inland Fisheries.

### Bird capture and housing

We captured 25 adult house sparrows (10 females, 15 males) at various locations around Blacksburg, VA, USA (37.21 N, 80.43 W, ∼700 m above sea level) from February 8^th^ to the 15^th^, 2013. Upon capture, we banded birds using a numbered aluminum ring, measured their tarsus (to the nearest 0.1 mm) and weighed them (body mass to the nearest 0.25 g). Immediately following capture, we took birds to an indoor facility, separated them into individual cages (31×41×41 w×l×h cm) within a single room, and placed them on a 13∶11 D∶L cycle that approximately matched the natural photoperiod at the time of capture. For one week following capture, we fed birds a commercial seed mixture *ad libitum*. Water, cuttlefish bone, and fine grit also were provided *ad libitum* as a source of calcium and to facilitate digestion. Because the experimental protocol required a daily change of food and water, a small cardboard box (approximately 18×10×15 w×l×h cm) was provided in every cage as a shelter from human disturbance and for roosting. Bird care was always provided at the same time of day (1300 h).

### Measuring daily food intake

After one week of acclimation, we measured the daily food intake for each individual for five consecutive days. The mass (±0.01 g) and the volume (±0.1 mL) of the food were highly correlated (adjusted R^2^ = 0.98, p<0.001); therefore, we used volume to measure food intake. Individuals received a daily food volume of 20 mL (approximately 12.9 g) in their feeder. The next day (24 hours later), we removed the bottom of the cage and the feeder for each individual cage, removed the discarded seed husks, and measured the remaining volume and the amount of food spilled with a graduated cylinder. The daily amount of food consumed was calculated as the difference between the initial volume in the feeder and the remaining volume in the feeder plus the spillage. “Daily food intake” was calculated as the median of the 5-day daily food intake values. Average daily food intake was 7.7±0.3 (SE) mL (range: 5.0–10.4).

### Experimental protocol

Following individual food intake measurements, individuals were randomly assigned to one of two identical rooms to reduce the disturbance caused by human presence during feeding and blood sampling. We provided all individuals with 110% of their daily food intake for one week as an additional acclimation period and then started the five-week experimental protocol. In each room, the birds were randomly assigned to one of the treatments: food restricted or control (60% or 110% of their individual daily food intake, respectively). The amount of food restriction was chosen because 60% of daily food intake was sufficient to induce increased corticosterone secretion (as opposed to the beneficial effects of slight calorie restriction) in two previous captive studies, although in different species [17], [51]. To facilitate distribution of the required amount of food, we made two containers corresponding to the volume of each individual's 110% and 60% daily food intake. Each treatment lasted for one week, and the treatments were alternated such that each individual experienced food restricted and control diets twice, but on week one, 12 birds started with the food-restricted treatment whereas 13 started with the control treatment. After week 2, all birds were given a recovery week *(ad libitum* food, enriched with chopped hard boiled eggs, vitamin supplements, cuttlefish bone, and grit). At the end of each week, we recorded the body mass of the birds and took blood samples (within 3 minutes of disturbance) for hormone and oxidative stress analysis (see below). Blood samples were not taken after the recovery week (week 3). Five birds died during the experiment (two birds during week 1, and three during week 2 – all of them during the food restriction treatment), so hormonal plasticity could be estimated for only the twenty surviving birds. We refer to the first part of the experiment (weeks 1-2) as replicate 1 and the second part of the experiment (week 4–5) as replicate 2.

### Blood sampling

To obtain initial blood samples for all birds, we staggered the start of the experiment so that each day there were at most four birds sampled from each room. We randomized the order in which the birds started the treatments, so that each day an equal number of birds received the food restricted and the control treatment. The order of blood sampling (with respect to room and then cage number) was also randomized, and several people bled the birds in a given room simultaneously. We took a blood sample within three minutes from the time when the first person entered the room. We refer to these samples as initial blood samples. Bleeding was always carried out at 13:00, and all birds were fed immediately after blood sampling. Blood was then stored on ice and centrifuged within 20 mins for 10 mins. Separated plasma was frozen at −20°C until the assay was performed.

### Hormone assay

Total corticosterone from plasma samples was quantified at Virginia Tech through direct radioimmunoassay, as described in detail in [52], [53]. Briefly, corticosterone was extracted from 15 µL plasma using dichloromethane, and extracts were reconstituted in PBS buffer. Mean recovery of corticosterone was 67% and final concentrations were corrected for individual recoveries. We used a commercial antiserum (Esoterix Endocrinology, Calabasas Hills, CA 91301, Product number: B3-163). The extracts were incubated overnight at 4°C with 14 K dpm (10 K cpm) of ^3^H-Corticosterone (Perkin Elmer, Product number: NET399250UC) and antiserum. Corticosterone bound to antibodies was separated by adding dextran-coated charcoal. After centrifugation and decanting, the radioactivity of the bound fraction was counted in a liquid scintillation counter. Within-assay variation among replicate known-concentration standard samples was 5.0% (N = 5 standards). Minimal detectable corticosterone levels were 1.12 ng/mL, and no samples fell below this detection limit.

### Oxidative stress assays

We assessed reactive oxygen metabolites (ROM) and total plasma antioxidant capacity (TAC), using the d-ROMs and OXY-adsorbent tests (Diacron International, Grosseto, Italy) respectively, in all blood samples as described previously [20], [54]. Oxidative damage to DNA was determined using an ELISA specific to three oxidized forms of the nucleic acid guanine, in both DNA and RNA (Cayman Chemical Company, Ann Arbor, MI, USA) and is expressed as the levels of 8-hydroxy-2′-deoxyguanosine (mg/mL) in the plasma sample. This assay was optimized for house sparrows, so that plasma samples were diluted to a concentration of 1∶100 and plated with an acetylcholinesterase monoclonal antibody and tracer. In all three assays, samples were assayed in duplicate and absorbance was measured in a microplate reader per manufactures specifications (BioTek ELx800). Intra-assay coefficients of variation were 3.9% for DNA damage, 4.5% for ROM, and 4.5% for TAC. TAC was expressed as mM of HClO neutralized, and ROM shows the total oxidant capacity (mainly due to hydroperoxide) of plasma samples against the chromogenic substrate N,N-diethylparaphenylendiamin, and is expressed in mM of H_2_O_2_ equivalents).

### Statistical analysis

All statistical analyses were performed in the R statistical and computing environment, version 3.0.1. [55] using Bayesian mixed-effects models based on Markov chain Monte Carlo estimations, as implemented in the R-package MCMCglmm version 2.17 [56], [57]. MCMCglmm allows the simultaneous analysis of multiple response variables (in our case: corticosterone and body mass or oxidative stress parameters) while controlling for the repeated measures of the response. This procedure makes it possible to analyse the between-individual differences in plasticity and also to analyse whether the measure of plasticity is related to another variable in a single model, while avoiding the problems associated with the usage of correlations with estimates derived from another statistical model [9], [58], [59]. First, to investigate individual differences in the reaction norms and the intercept-slope covariance, we fitted a MCMCglmm model with corticosterone as a response variable, and treatment and the order of treatments (i.e., treatment on week 1) as fixed factors. The random structure of the model was changed to test different hypotheses about individual variation in the intercept and the slopes of the reaction norms. Second, to test whether hormonal responsiveness was related to body mass and to the levels of oxidative stress, we used bivariate MCMCglmm models to investigate the covariance between the two response variables (corticosterone and either body mass or oxidative damage to DNA). Corticosterone values were log-transformed before analyses. In bivariate models, the response variables were standardized (i.e., their mean was 0 and their SD was 1). We compared candidate models using the information-theoretic approach [60]. Specifically, we used the Deviance Information Criterion (DIC) [57], which is the Bayesian equivalent of the more commonly used Akaike Information Criterion. Initial analyses showed that neither sex, nor the room the animals were housed in had any detectable effect on our results; therefore, these factors were excluded from the final analyses. We report the parameter estimates (posterior means of treatment compared to control) and corresponding 95% Credibility Interval values in brackets, i.e. mean [lower 95% CI; upper 95% CI]. We used inverse Wishart priors, (a weakly informative prior, which allows for minimal influence of the prior on the posterior distributions), and a residual variance structure separately for treatments. We ran the models three times and then assessed the convergence of the models using the Gelman-Rubin statistic implemented in the coda package in R (functions gelman.diag and gelman.plot) [61]. We report statistical results for the last run of the MCMC chains.

## Results

### Effects of treatment

**Figure 2:**
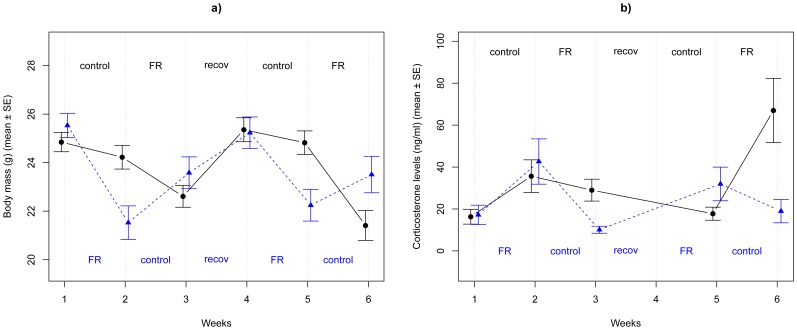
Mean ± SE (a) body mass (b) corticosterone levels during the experiment. Birds received either a food restricted ('FR') or a control diet, which corresponded to 60% or 110% of their daily food consumption, respectively. During the third week of the experiment, all birds received *ad libitum* food for one week ('recov'). Birds were randomly allocated into two groups that differed only in the order of the treatments. Black dots and solid lines represent birds starting with the control diet (treatments indicated above the lines), blue triangles and dotted lines represent birds starting with the food restricted diet (treatments indicated below the lines). Note that blood samples were not collected after the recovery period (after week 3), but body mass was recorded.

Birds assigned to the food restricted or control diet on week 1 did not differ in their body size (tarsus length) or body mass at capture (Table S1). At the beginning of the experiment (after the acclimation period, week 1) there was no difference in body mass, corticosterone levels, or any of the oxidative parameters (Table S1, see also Fig. 2). As expected, diet treatment affected body mass: during food restriction birds lost an average 9% of their body mass (−2.17 [−2.56; −1.74] g, Fig. 2a).

Changes in body mass were associated with changes in corticosterone levels: overall, during food restriction, corticosterone levels were doubled compared to the control periods (96%, 30.19 [16.15; 57.81] ng/mL increase, Fig. 2b). The two groups (receiving different diets on week 1) differed in their overall corticosterone response to the treatment: birds starting with food restriction had overall lower corticosterone levels (−8.36 [−11.76; −2.59] ng/mL, Fig. 2b). We controlled for this effect in the fixed structure of the subsequent models. However, on average, corticosterone levels of the birds during replicate 1 (week 1–2) and replicate 2 (week 4–5) did not differ (−0.31 [−5.66; 6.39] ng/mL).

Levels of oxidative damage to DNA and ROM were unaffected by the treatment (damage: −1.85 [−5.49; 2.47] mg/mL, ROM: 1.36 [−3.43; 5.92] mM). However, total antioxidant capacity was lower during food restriction (−22.46 [−39.79; −2.58] mM).

### Individual differences in corticosterone reaction norms

#### Is there individual variation in initial corticosterone levels?

The model including individual as a random factor had better support than the constrained model that provides a common estimate for initial corticosterone (random intercept model: DIC  = 196.28, constrained model: DIC  = 199.23). The difference between these models supports the existence of consistent individual variation and shows that individuals differ in their initial corticosterone levels.

#### Is there individual variation in plasticity of corticosterone levels (i.e. reaction norm slope)?

**Figure 3:**
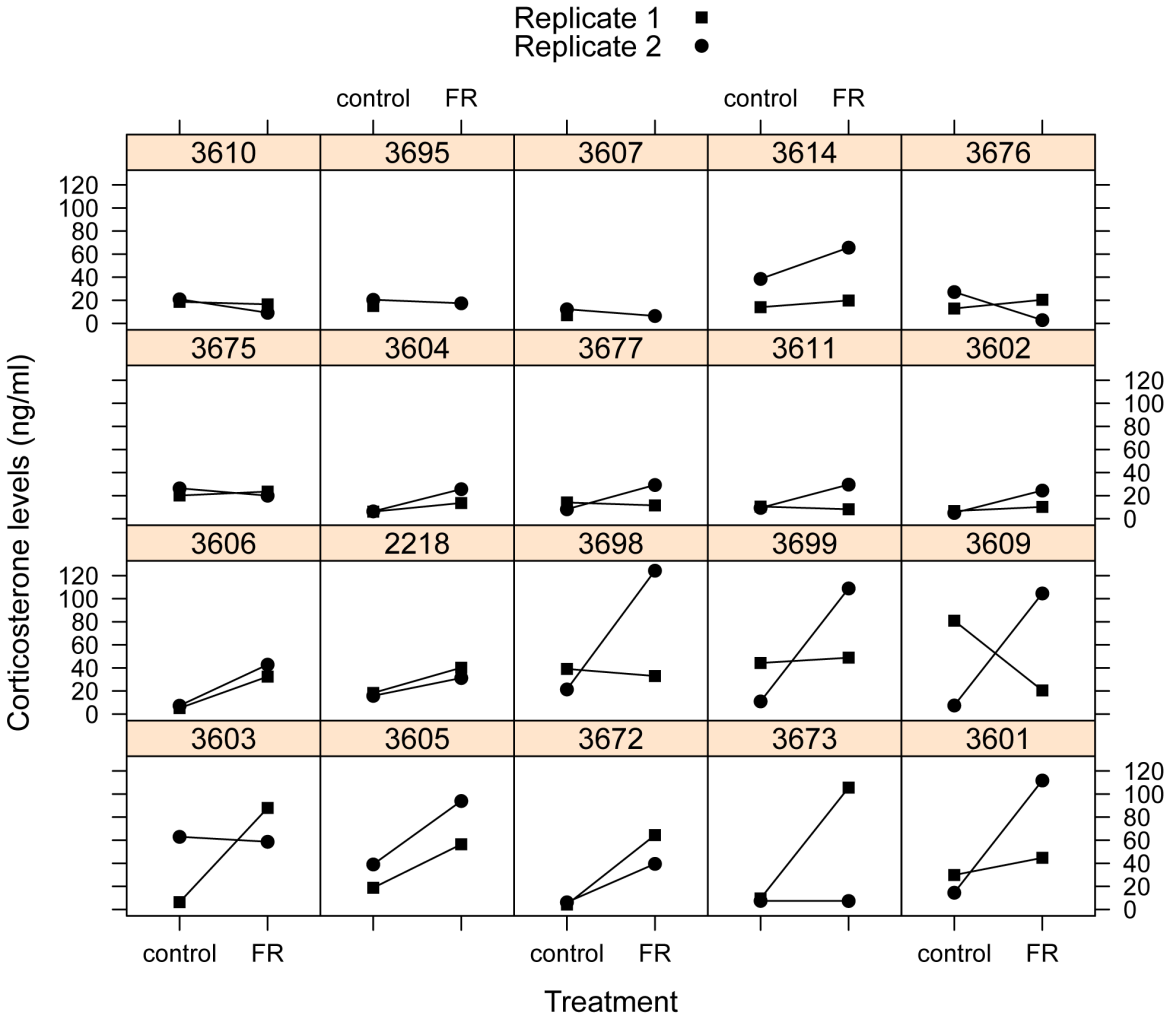
Corticosterone levels in response to a dietary treatment in house sparrows. The treatment was either control or food restricted (‘FR’), which corresponded to 110% or 60% respectively of daily food intake for each individuals. Each individual (denoted here by their band numbers above each box) received both treatments twice during the course of the study. Replicate 1 or 2 refers to the weeks (1–2 and 4–5 respectively) when they received the treatments. One blood sample was missed for two individuals (3607, 3695 in the top row). Individuals are ordered by degree of plasticity (after controlling for changes in their body mass), as in Fig.4.

The model including individual variation in responsiveness received better support than the model with common slopes across individuals, but the difference was small (ΔDIC  = −0.51, Fig. 3). However, a single individual (3609) had an unusually high corticosterone level (80.9 ng/mL) during the first control treatment leading to a negative slope in the first replicate (Fig. 3) and a disproportionate effect on the model fit. After removing this single outlier (3.85 times above the upper quartile of corticosterone levels in the control treatment), the difference between the random intercept and the random intercept + random slope model became more pronounced (ΔDIC  = −2.37, model 2 vs. model 4 in Table 1). The latter model included a covariance term between responsiveness and initial levels (i.e., reaction norm slope and intercept). In the next step, we constrained the model by setting the slope-intercept covariance to zero. This model received slightly better support as the model where slope-intercept covariance was estimated (ΔDIC  = −0.43, Table 1). Finally, we fitted a model where the covariance between slope and the intercept was constrained to be zero, and they shared a common variance term. This model received similar support as the random intercept model (Table 1). Therefore, we found robust differences in corticosterone responsiveness (plasticity) between individuals, and weak support for a lack of covariance between initial corticosterone levels and responsiveness.

**Table 1:** Comparison of *a priori* candidate models of individual differences in corticosterone responsiveness.

id	Random effect	Model description	K	DIC	ΔDIC
3	∼idh(treatment):band	random intercept and slope, no covariance between slope and intercept	6	176.1	0.000
2	∼us(treatment):band	random intercept and slope, covariance between slope and intercept	6	176.5	0.430
1	∼band	random slope only	5	178.9	2.796
4	∼treatment:band	same variance for intercept and slope, no covariance	5	179.4	3.318

The variables under the column “Random effect” indicate the specific random effect in the model in which “band” corresponds to the individual. K is the number of parameters in the model, DIC is deviance information criterion, ΔDIC indicates the difference between DIC value of a given model and the model with the lowest DIC. All models were fit with treatment as a fixed effect in the model; therefore, models differ in their random structure.

#### How much of the variation in corticosterone levels is explained by changes in body mass?

**Figure 4:**
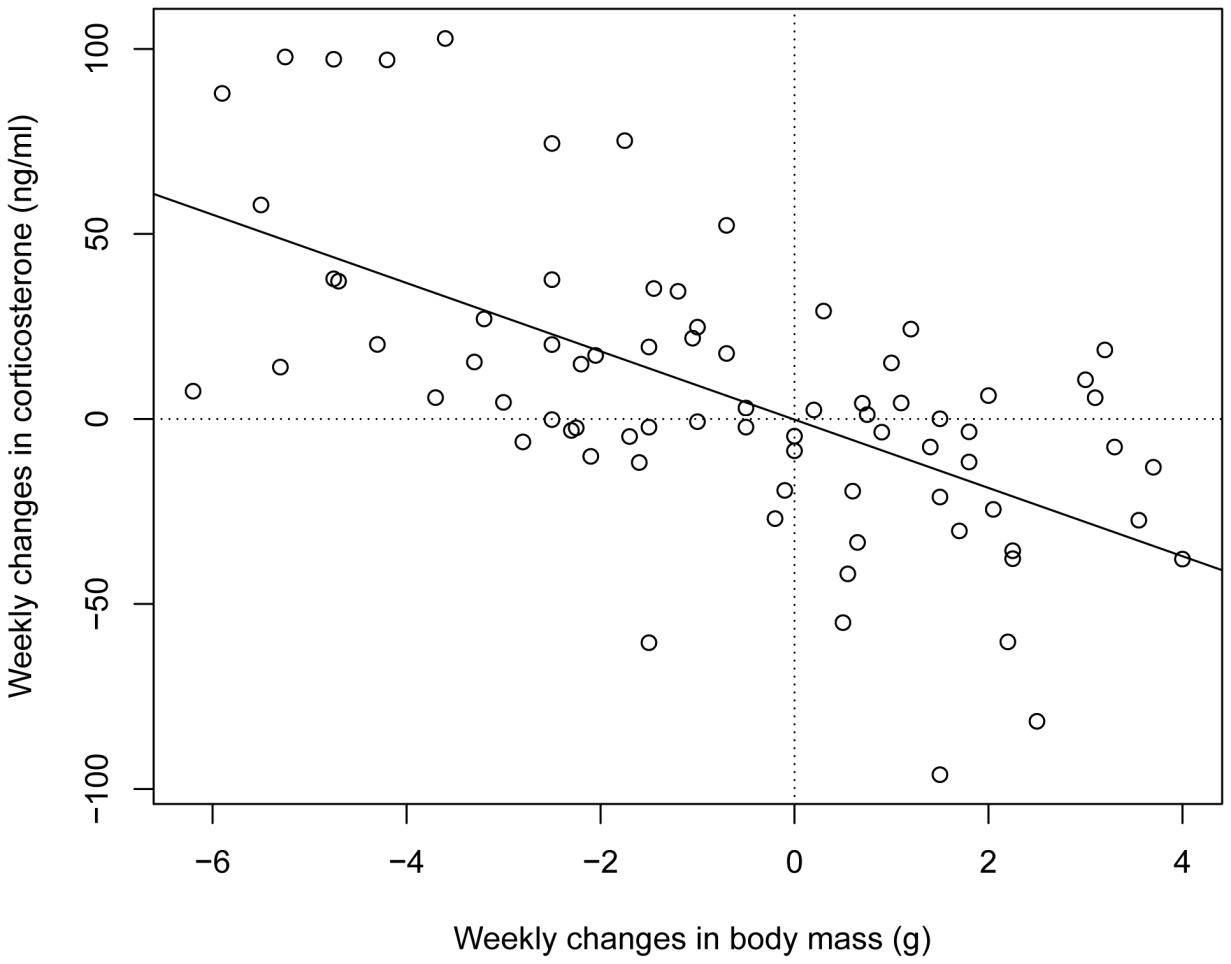
Relationship between the weekly changes in body mass and corticosterone levels in all experimental house sparrows. Note that individuals are represented with multiple points. The line represents the model fit of the fixed model that accounts for the repeated nature of the data.

Overall, changes in corticosterone levels were negatively associated with changes in body mass: on average, birds reduced their circulating corticosterone levels by 9.31 [6.96; 12.35] ng/mL for every one gram increase in their body mass. Remarkably, the intercept was almost exactly zero (−0.14 [−6.84; 7.12] ng/mL), indicating that on average, loss of body mass was associated with increases in corticosterone, whereas gain in body mass was followed by a decrease in corticosterone (Fig. 4).

Although this relationship between changes in body mass and corticosterone suggests a within-individual correlation, we also tested whether the variation among individuals in their body mass may affect our results (e.g., because heavier birds may lose more mass). Therefore, we analyzed further how body mass affects corticosterone levels by partitioning the variance in body mass into within-individual and between-individual components using within-individual centering [62]: for each individual we calculated the mean body mass (between-individual variance component) and the differences from its own mean (within-individual variance component). This analysis showed that variation in corticosterone levels was caused mainly by within-individual variation in body mass: in general, the more mass a bird lost over a week, the higher its corticosterone levels (intercept (log): 4.36, within-individual effect of mass: −0.28 [−0.39; −0.16]), while the between-individual effect of mass was weak: −0.06 [−0.19; 0.06]).

**Figure 5:**
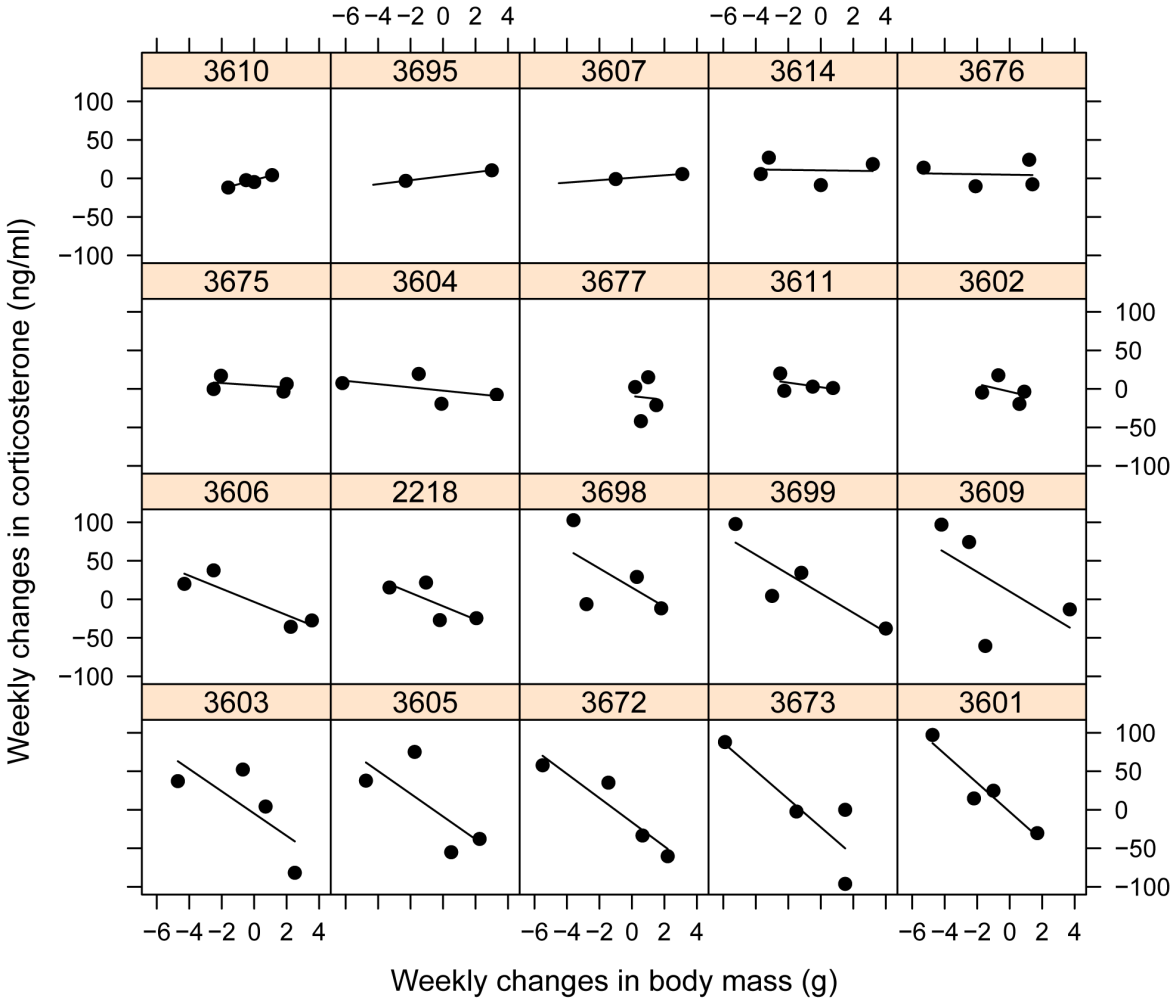
Relationship between the weekly changes in body mass and corticosterone levels in individual house sparrows. Individuals are denoted by their band number above each box. Panels are ordered in function of the individuals' conditional responsiveness (i.e., the slope of the fitted line), from left to right starting with the top row.

Therefore, we controlled for the individual variation in body mass and re-analyzed the individual differences in corticosterone responsiveness. To do so, we analyzed body mass and corticosterone levels in a bivariate Bayesian mixed model, and we calculated a conditional between-individual variance (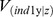) in corticosterone response using the equation
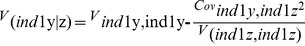
(1)where *ind*1*y* and *ind*1*z* refers to the reaction norm slope in corticosterone and in mass, respectively, and *V* and *Cov* denote variance and covariance terms respectively. In other words, 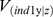 represents the differences in responsiveness between individuals that are independent of the body mass changes induced by the treatment [9]. We found significant between-individual differences in corticosterone responsiveness even after controlling for the effects of body mass change (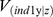  = 0.61 [0.2; 1.04], Fig. 5).

Although between-individual variation in body mass did not affect corticosterone levels (see above), daily food consumption during *ad libitum* circumstances before the experiment predicted individual conditional plasticity: on average, one gram increase in daily food consumed was associated with 2.44 [0.42; 4.47] ng/ml decrease in corticosterone levels for one gram loss of body mass.

### Costs of hormonal plasticity

#### Is corticosterone responsiveness related to oxidative stress?

Corticosterone responsiveness was unrelated to total antioxidant capacity, ROM levels or the oxidative damage to DNA suffered during the experiment, as shown by the lack of covariance between corticosterone reaction norm slope and the above oxidative parameters (TAC: −0.18 [−0.61; 0.16], ROM: 0.02 [−0.43; 0.47], damage: −0.12 [−0.59; 0.25]).

## Discussion

Our study revealed significant differences in the way that individuals responded to a standardized dietary treatment: individuals differed in both aspects of their corticosterone reaction norms: their initial levels and their responsiveness. To the best of our knowledge, these results are the first to specifically address and document substantial individual differences in both the elevation and the slope of the corticosterone reaction norms within a population. These individual by environment (I×E) differences can have important consequences for the evolution of physiological mechanisms by which individuals cope with environmental challenges.

We propose two explanations for why these differences in the birds' hormonal reaction norm may exist. First, it is possible that individuals did not perceive the food restriction as equally challenging. When we investigated the effects of food restriction on all birds, we found that the treatment was overall effective, such that food deprivation resulted in considerable loss of body mass and strongly elevated corticosterone levels (Fig. 2), indicative of a ‘stress-response' [63]–[65]. Despite these robust effects of the treatment, our study revealed important individual variation in how individuals responded to the treatment (Fig. 3) that raises the possibility that even if we standardized the treatment for each individual, we may not have been able to achieve equally challenging conditions for all birds. Inevitable errors in determining the daily food intake, or simply large within-individual variation in food intake, may have caused variation in the severity of the food restriction. However, our results are not consistent with this explanation. When we controlled for the within-individual variation in body mass changes, the conditional between-individual variance in corticosterone responsiveness was still evident. In other words, even after controlling for differences in changes in body mass, individuals still differed in the way their corticosterone levels responded to the treatment (for example, compare bird 3672 and 3676 in Fig. 3 and in Fig. 5). Nevertheless, we cannot exclude the possibility that intrinsic differences in the birds' metabolism may make some individuals more tolerant of food shortages and changes in body mass. In fact, even subtle variation in metabolism may generate consistent individual differences in a variety of behavioral and physiological traits [66]. Our result that initial food consumption was related to conditional plasticity seems consistent with this explanation. Also, the order of the treatments seemed to affect the way birds responded to the treatments. When birds received the control treatment after the *ad libitum* periods, they lost mass and increased their corticosterone levels, whereas birds that received the control treatment after the food restriction were able to recuperate and gain back mass and decrease corticosterone levels during the control period. This result suggests that prior conditions could affect perception and/or response to subsequent environmental stressors.

A second, mutually non-exclusive explanation for the existence of different reaction norms is that even in the face of equally perceived challenges, individuals genuinely differ in their coping strategies. Consistent individual differences in corticosterone secretion have been found in several species [23], [25], [27], [67], [68]. In our study, individuals differed not only in their initial corticosterone levels (reaction norm elevation), but also in their responsiveness (i.e., reaction norm slope, or plasticity). Our results do not allow us to reject the hypothesis that these two aspects of the reaction norm are related (our models with and without the intercept-slope covariance received roughly equal support, Table 1), indicating that responsiveness may not be limited or constrained by higher initial corticosterone levels, or if there is such an effect, it is weak.

What is the functional cost of these between-individual differences in physiological responsiveness? None of the measured oxidative stress parameters (reactive oxygen metabolites, the amount of DNA damage or the total antioxidant capacity) were related to the individual differences in hormonal responsiveness. Among these variables, total antioxidant capacity varied most with the treatment, and was significantly lower during food restriction than during control diet, but even this variable was not related to the individual variation in corticosterone. These results present an interesting conundrum.

One possibility is that individual differences in stress responsiveness are not related to fitness. This explanation seems unlikely, because previous correlative and experimental work have shown that the stress response is related to survival and reproductive success, although these studies used the capture-restraint stressor (e.g. [69]–[71]). Also, we have to differentiate between costs of the hormonal response itself and the costs of the associated behavioral and physiological changes the hormonal response can mediate [72], [73]. Even if producing large amounts of corticosterone is effectively cost-free, it does not necessarily mean that these differences in hormone secretion are not translated into fitness differences [72]. In this study, we measured surrogate measures of tissue integrity and repair, but it is possible that stress responsiveness affects other fitness-related traits that were not measured in the current study [74]. For example, we only measured oxidative stress parameters from the blood, although oxidative damage may be more readily detected from tissues, such as the brain or the liver [49]. Previous studies have shown that corticosterone response to capture stress may be related to both reproduction and survival, although these effects are often context-dependent [21], [70], [75]–[78]. Also, as we discussed above, individual differences in the perceived severity of the treatment may have affected oxidative stress parameters. Calorie restriction is known to have a beneficial effect on oxidative balance [79]. If calorie restriction resulted in an overall improvement of oxidative balance, it could have uncoupled the link between corticosterone responsiveness and the oxidative stress parameters.

Between-individual differences in responsiveness might also be part of the stable behavioral and physiological trait complexes of the individuals, often referred to as ‘personality’ or ‘coping styles’ [80]. For instance, artificial selection for different stress responsiveness in rainbow trout and zebra finches was associated with concurrent changes in behavior, physiology, and central signaling systems [28], [30]. Although the artificial selection studies have shown that there is a genetic basis of some of the individual differences in stress responsiveness, we also know that this trait itself shows developmental plasticity. Environment (including maternal effects) during early development may significantly influence the coping style of the adult [19], [81]–[84]. This developmental plasticity of physiological responsiveness plays an important role in producing phenotypes that match the predicted environment in later life stages. These genetic and developmental effects may create variability in coping styles in a population, while frequency dependent selection and spatio-temporal heterogeneity in the environment may ensure the persistence of these different strategies [85]–[87]. Irrespective of the actual causes of the variation, the fact that we found substantial individual differences in a relatively small sample of wild birds is not consistent with the idea of strong selection favoring a single optimal reaction norm. Individuals seemed to fall into two main categories: those that do not show a substantial increase in corticosterone levels in response to the treatment (‘non responders’: top two rows in Fig. 5) and those that do (‘responders’ bottom two rows in Fig. 5, see also Fig. S1). Although a larger sample is needed to test whether the distribution of responsiveness at the population level is indeed bimodal, the pattern in our sample is consistent with the existence of alternative coping styles.

The existence of multiple reaction norms (as represented by a significant individual – environment interaction, I×E) in a population has interesting evolutionary and ecological implications: if individuals do not experience all environments throughout their lifetime, then natural selection is masked at the intersection of the reaction norms, because different genotypes produce the same phenotype in this range [4]. In this context, phenotypic plasticity has been traditionally considered to constrain or slow evolutionary responses [13]. However, recent empirical and theoretical work has shown that adaptive plasticity contributes to the maintenance of genetic variation within populations, reduces bottlenecks when facing rapid environmental changes, and confers an overall faster rate of adaptation, which is very relevant in face of recent global changes [59], [88]–[90].

Finally, our results also serve as a cautionary note about the interpretation of phenotypic correlations among labile traits. Similarly to the findings of another study [17], we found a general negative relationship between corticosterone levels and body mass. The repeated measures design of our experiment allowed us to tease apart within- and between-individual effects, and we showed that the relationship between body mass and corticosterone was caused by within-individual effects, and not between-individual effects (i.e., not because heavier birds had overall lower corticosterone levels than lighter birds). It is noteworthy, because changes in labile phenotypic traits within individuals may account for a large part, or even all of the phenotypic variance in a population. Without recognizing the latter, researchers may be tempted to document evidence for natural selection [5], [7], [8] even when such conclusions are based on phenotypic correlations that result largely from within-individual sources [91]. To avoid that pitfall, we recommend studies where individuals are repeatedly sampled within and across environments.

## Supporting Information

Figure S1 (TIF)

Table S1 (DOCX)

## References

[1] Bennett AJ (2008) Gene environment interplay: Nonhuman primate models in the study of resilience and vulnerability. Dev Psychobiol 50: 48–59 10.1002/dev.20263 18085557

[2] Williams TD (2008) Individual variation in endocrine systems: moving beyond the “tyranny of the Golden Mean.”. Philos Trans R Soc B Biol Sci 363: 1687–1698 10.1098/rstb.2007.0003 PMC2606721 18048295

[3] Dochtermann NA, Dingemanse NJ (2013) Behavioral syndromes as evolutionary constraints. Behav Ecol 24: 806–811 10.1093/beheco/art002

[4] Pigliucci M (2001) Phenotypic Plasticity: Beyond Nature and Nurture. JHU Press. 356 p.

[5] McGlothlin JW, Whittaker DJ, Schrock SE, Gerlach NM, Jawor JM, et al (2010) Natural Selection on Testosterone Production in a Wild Songbird Population. Am Nat 175: 687–701 10.1086/648324 20394524

[6] Crespi EJ, Williams TD, Jessop TS, Delehanty B (2013) Life history and the ecology of stress: how do glucocorticoid hormones influence life-history variation in animals? Funct Ecol 27: 93–106 10.1111/1365-2435.12009

[7] Ouyang JQ, Sharp P, Quetting M, Hau M (2013) Endocrine phenotype, reproductive success and survival in the great tit, Parus major. J Evol Biol 26: 1988–1998 10.1111/jeb.12202 23961922

[8] Patterson SH, Hahn TP, Cornelius JM, Breuner CW (2014) Natural selection and glucocorticoid physiology. J Evol Biol 27: 259–274 10.1111/jeb.12286 24341364

[9] Dingemanse NJ, Dochtermann NA (2013) Quantifying individual variation in behaviour: mixed-effect modelling approaches. J Anim Ecol 82: 39–54 10.1111/1365-2656.12013 23171297

[10] Dingemanse NJ, Kazem AJ, Réale D, Wright J (2010) Behavioural reaction norms: animal personality meets individual plasticity. Trends Ecol Evol 25: 81–89 10.1016/j.tree.2009.07.013 19748700

[11] MacDougall-Shackleton SA, Schmidt KL, Furlonger AA, MacDougall-Shackleton EA (2013) HPA axis regulation, survival, and reproduction in free-living sparrows: Functional relationships or developmental correlations? Gen Comp Endocrinol 190: 188–193 10.1016/j.ygcen.2013.05.026 23770216

[12] Bonier F, Martin PR, Moore IT, Wingfield JC (2009) Do baseline glucocorticoids predict fitness? Trends Ecol Evol 24: 634–642 10.1016/j.tree.2009.04.013 19679371

[13] Ghalambor CK, McKAY JK, Carroll SP, Reznick DN (2007) Adaptive versus non-adaptive phenotypic plasticity and the potential for contemporary adaptation in new environments. Funct Ecol 21: 394–407 10.1111/j.1365-2435.2007.01283.x

[14] Dingemanse NJ, Edelaar P, Kempenaers B (2010) Why is there variation in baseline glucocorticoid levels? Trends Ecol Evol 25: 261–262 10.1016/j.tree.2010.01.008 20144490

[15] Bonier F, Martin PR, Moore IT, Wingfield JC (2010) Clarifying the Cort-Fitness Hypothesis: a response to Dingemanse et al. Trends Ecol Evol 25: 262–263 10.1016/j.tree.2010.01.009

[16] Breuner CW, Wingfield JC, Romero LM (1999) Diel rhythms of basal and stress-induced corticosterone in a wild, seasonal vertebrate, Gambel's white-crowned sparrow. J Exp Zool 284: 334–342 doi:10.1002/(SICI)1097-010X(19990801)284:3<334::AID-JEZ11>3.0.CO;2-# 10404125

[17] Fokidis HB, Hurley L, Rogowski C, Sweazea K, Deviche P (2011) Effects of Captivity and Body Condition on Plasma Corticosterone, Locomotor Behavior, and Plasma Metabolites in Curve-Billed Thrashers. Physiol Biochem Zool 84: 595–606 10.1086/662068 22030852

[18] Ouyang JQ, Sharp PJ, Dawson A, Quetting M, Hau M (2011) Hormone Levels Predict Individual Differences in Reproductive Success in a Passerine Bird. Proc R Soc B Biol Sci 278: 2537–2545 10.1098/rspb.2010.2490 PMC3125628 21247953

[19] Love OP, Williams TD (2008) Plasticity in the adrenocortical response of a free-living vertebrate: the role of pre- and post-natal developmental stress. Horm Behav 54: 496–505 10.1016/j.yhbeh.2008.01.006 18313054

[20] Haussmann MF, Longenecker AS, Marchetto NM, Juliano SA, Bowden RM (2012) Embryonic Exposure to Corticosterone Modifies the Juvenile Stress Response, Oxidative Stress and Telomere Length. Proc R Soc B Biol Sci 279: 1447–1456 10.1098/rspb.2011.1913 PMC3282378 22072607

[21] Lendvai ÁZ, Giraudeau M, Chastel O (2007) Reproduction and modulation of the stress response: an experimental test in the house sparrow. Proc R Soc BBiol Sci 274: 391 10.1098/rspb.2006.3735 PMC1702373 17164203

[22] Love OP, Madliger CL, Bourgeon S, Semeniuk CAD, Williams TD (2014) Evidence for baseline glucocorticoids as mediators of reproductive investment in a wild bird. Gen Comp Endocrinol 199: 65–69 10.1016/j.ygcen.2014.01.001 24462764

[23] Rensel MA, Schoech SJ (2011) Repeatability of baseline and stress-induced corticosterone levels across early life stages in the Florida scrub-jay (Aphelocoma coerulescens). Horm Behav 59: 497–502 10.1016/j.yhbeh.2011.01.010 21295036

[24] Wilcoxen TE, Boughton RK, Bridge ES, Rensel MA, Schoech SJ (2011) Age-related differences in baseline and stress-induced corticosterone in Florida scrub-jays. Gen Comp Endocrinol 173: 461–466 10.1016/j.ygcen.2011.07.007 21827761

[25] Cockrem JF (2013) Individual variation in glucocorticoid stress responses in animals. Gen Comp Endocrinol 181: 45–58 10.1016/j.ygcen.2012.11.025 23298571

[26] Cockrem JF, Candy EJ, Castille SA, Satterlee DG (2010) Plasma corticosterone responses to handling in Japanese quail selected for low or high plasma corticosterone responses to brief restraint. Br Poult Sci 51: 453–459 10.1080/00071668.2010.503637 20680881

[27] Cockrem JF, Barrett DP, Candy EJ, Potter MA (2009) Corticosterone responses in birds: Individual variation and repeatability in Adelie penguins (Pygoscelis adeliae) and other species, and the use of power analysis to determine sample sizes. Gen Comp Endocrinol 163: 158–168 10.1016/j.ygcen.2009.03.029 19351536

[28] Evans MR, Roberts ML, Buchanan KL, Goldsmith AR (2006) Heritability of corticosterone response and changes in life history traits during selection in the zebra finch. J Evol Biol 19: 343–352 10.1111/j.1420-9101.2005.01034.x 16599910

[29] Narayan EJ, Cockrem JF, Hero J-M (2013) Are baseline and short-term corticosterone stress responses in free-living amphibians repeatable? Comp Biochem Physiol A Mol Integr Physiol 164: 21–28 10.1016/j.cbpa.2012.10.001 23047053

[30] Øverli Ø, Winberg S, Pottinger TG (2005) Behavioral and Neuroendocrine Correlates of Selection for Stress Responsiveness in Rainbow Trout—a Review. Integr Comp Biol 45: 463–474 10.1093/icb/45.3.463 21676791

[31] Jenkins BR, Vitousek MN, Hubbard JK, Safran RJ (2014) An experimental analysis of the heritability of variation in glucocorticoid concentrations in a wild avian population. Proc R Soc B Biol Sci 281: 20141302 10.1098/rspb.2014.1302 PMC4123711 25056627

[32] Lendvai ÁZ, Bókony V, Angelier F, Chastel O, Sol D (2013) Do smart birds stress less? An interspecific relationship between brain size and corticosterone levels. Proc R Soc B Biol Sci 280: 20131734 10.1098/rspb.2013.1734 PMC3779331 24026820

[33] Wingfield JC (1994) Modulation of the adrenocortical response to stress in birds. Perspectives in comparative endocrinology. National Research Council of Canada. pp. 520–528.

[34] Schoech SJ, Romero LM, Moore IT, Bonier F (2013) Constraints, concerns and considerations about the necessity of estimating free glucocorticoid concentrations for field endocrine studies. Funct Ecol 25: 1100–1106 10.1111/1365-2435.12142

[35] Buck CL, O′Reilly KM, Kildaw SD (2007) Interannual variability of Black-legged Kittiwake productivity is reflected in baseline plasma corticosterone. Gen Comp Endocrinol 150: 430–436 10.1016/j.ygcen.2006.10.011 17161400

[36] Kitaysky AS, Wingfield JC, Piatt JF (2001) Corticosterone facilitates begging and affects resource allocation in the black-legged kittiwake. Behav Ecol 12: 619–625 10.1093/beheco/12.5.619

[37] Wingfield JC, Sapolsky R (2003) Reproduction and Resistance to Stress: When and How. J Neuroendocrinol 15: 711–724 10.1046/j.1365-2826.2003.01033.x 12834431

[38] Monaghan P, Metcalfe NB, Torres R (2009) Oxidative stress as a mediator of life history trade-offs: mechanisms, measurements and interpretation. Ecol Lett 12: 75–92 10.1111/j.1461-0248.2008.01258.x 19016828

[39] Metcalfe NB, Alonso-Alvarez C (2010) Oxidative stress as a life-history constraint: the role of reactive oxygen species in shaping phenotypes from conception to death. Funct Ecol 24: 984–996 10.1111/j.1365-2435.2010.01750.x

[40] Hill GE (2011) Condition-dependent traits as signals of the functionality of vital cellular processes. Ecol Lett 14: 625–634 10.1111/j.1461-0248.2011.01622.x 21518211

[41] Selman C, Blount JD, Nussey DH, Speakman JR (2012) Oxidative damage, ageing, and life-history evolution: where now? Trends Ecol Evol 27: 570–577 10.1016/j.tree.2012.06.006 22789512

[42] Costantini D (2008) Oxidative stress in ecology and evolution: lessons from avian studies. Ecol Lett 11: 1238–1251 10.1111/j.1461-0248.2008.01246.x 18803642

[43] Speakman JR, Garratt M (2014) Oxidative stress as a cost of reproduction: Beyond the simplistic trade-off model. BioEssays 36: 93–106 10.1002/bies.201300108 24285005

[44] Finkel T, Holbrook N (2000) Oxidants, oxidative stress and the biology of ageing. Nature 408: 239–247 10.1038/35041687 11089981

[45] Epel ES, Blackburn EH, Lin J, Dhabhar FS, Adler NE, et al (2004) Accelerated telomere shortening in response to life stress. Proc Natl Acad Sci U S A 101: 17312–17315 10.1073/pnas.0407162101 15574496 PMC534658

[46] Costantini D, Fanfani A, Dell′Omo G (2008) Effects of corticosteroids on oxidative damage and circulating carotenoids in captive adult kestrels (Falco tinnunculus). J Comp Physiol [B] 178: 829–835 10.1007/s00360-008-0270-z 18443799

[47] Epel ES (2009) Psychological and metabolic stress: a recipe for accelerated cellular aging? Horm Athens Greece 8: 7–22. 10.14310/horm.2002.1217 19269917

[48] Haussmann MF, Marchetto NM (2010) Telomeres: Linking stress and survival, ecology and evolution. Curr Zool 56: 714–727.

[49] Costantini D, Marasco V, Møller AP (2011) A meta-analysis of glucocorticoids as modulators of oxidative stress in vertebrates. J Comp Physiol B 181: 447–456 10.1007/s00360-011-0566-2 21416253

[50] Fokidis HB, Roziers MB des, Sparr R, Rogowski C, Sweazea K, et al (2012) Unpredictable food availability induces metabolic and hormonal changes independent of food intake in a sedentary songbird. J Exp Biol 215: 2920–2930 10.1242/jeb.071043 22837467

[51] Ottinger MA, Mobarak M, Abdelnabi M, Roth G, Proudman J, et al (2005) Effects of calorie restriction on reproductive and adrenal systems in Japanese quail: Are responses similar to mammals, particularly primates? Mech Ageing Dev 126: 967–975 10.1016/j.mad.2005.03.017 15935442

[52] Wingfield JC, Hegner RE, Lewis DM (1992) Hormonal responses to removal of a breeding male in the cooperatively breeding white-browed sparrow weaver, Plocepasser mahali. Horm Behav 26: 145–155 doi 10.1016/0018-506X(92)90038-W. 1612561

[53] Malueg AL, Walters JR, Moore IT (2009) Do stress hormones suppress helper reproduction in the cooperatively breeding red-cockaded woodpecker (Picoides borealis)? Behav Ecol Sociobiol 63: 687–698 10.1007/s00265-008-0702-5

[54] Treidel LA, Whitley BN, Benowitz-Fredericks ZM, Haussmann MF (2013) Prenatal exposure to testosterone impairs oxidative damage repair efficiency in the domestic chicken (Gallus gallus). Biol Lett 9: 20130684 10.1098/rsbl.2013.0684 24046877 PMC3971716

[55] R Core Team (2013) R: A language and environment for statistical computing. R Foundation for Statistical Computing, ISBN 3-900051-07-0. Available: http://www.R-project.org.

[56] Hadfield JD, Nakagawa S (2010) General quantitative genetic methods for comparative biology: phylogenies, taxonomies and multi-trait models for continuous and categorical characters. J Evol Biol 23: 494–508 10.1111/j.1420-9101.2009.01915.x 20070460

[57] Hadfield JD (2010) MCMC methods for multi-response generalized linear mixed models: the MCMCglmm R package. J Stat Softw 33: 1–22.20808728

[58] Brommer JE (2013) Phenotypic plasticity of labile traits in the wild. Curr Zool 59: 485–505.

[59] Dingemanse NJ, Wolf M (2013) Between-individual differences in behavioural plasticity within populations: causes and consequences. Anim Behav. Available: http://www.sciencedirect.com/science/article/pii/S0003347213000092. Accessed 2013 Feb 14

[60] Burnham KP, Anderson DR (2002) Model selection and multimodel inference: a practical information-theoretic approach. New York: Springer. 512 p.

[61] Plummer M, Best N, Cowles K, Vines K (2006) CODA: Convergence Diagnosis and Output Analysis for MCMC. R News 6: 7–11.

[62] Van de Pol M, Wright J (2009) A simple method for distinguishing within-versus between-subject effects using mixed models. Anim Behav 77: 753.

[63] Fokidis HB, Orchinik M, Deviche P (2009) Corticosterone and corticosteroid binding globulin in birds: relation to urbanization in a desert city. Gen Comp Endocrinol 160: 259–270 10.1016/j.ygcen.2008.12.005 19116155

[64] Romero LM, Cyr NE, Romero RC (2006) Corticosterone responses change seasonally in free-living house sparrows (Passer domesticus). Gen Comp Endocrinol 149: 58–65 10.1016/j.ygcen.2006.05.004 16774754

[65] Strochlic DE, Romero LM (2008) The effects of chronic psychological and physical stress on feather replacement in European starlings (Sturnus vulgaris). Comp Biochem Physiol A Mol Integr Physiol 149: 68–79 10.1016/j.cbpa.2007.10.011 18032078

[66] Biro PA, Stamps JA (2010) Do consistent individual differences in metabolic rate promote consistent individual differences in behavior? Trends Ecol Evol 25: 653–659 10.1016/j.tree.2010.08.003 20832898

[67] Romero LM, Reed JM (2008) Repeatability of baseline corticosterone concentrations. Gen Comp Endocrinol 156: 27–33 10.1016/j.ygcen.2007.10.001 18036526

[68] Baugh AT, Schaper SV, Hau M, Cockrem JF, de Goede P, et al (2012) Corticosterone responses differ between lines of great tits (Parus major) selected for divergent personalities. Gen Comp Endocrinol 175: 488–494 10.1016/j.ygcen.2011.12.012 22202603

[69] Groscolas R, Lacroix A, Robin J-P (2008) Spontaneous egg or chick abandonment in energy-depleted king penguins: A role for corticosterone and prolactin? Horm Behav 53: 51–60 10.1016/j.yhbeh.2007.08.010 17920597

[70] Angelier F, Holberton R, Marra P (2009) Does stress response predict return rate in a migratory bird species? A study of American redstarts and their non-breeding habitat. Proc R Soc Lond B 276: 3545–3551 10.1098/rspb.2009.0868 PMC2817190 19605397

[71] Romero LM, Wikelski M (2010) Stress physiology as a predictor of survival in Galapagos marine iguanas. Proc R Soc B Biol Sci 277: 3157–3162 10.1098/rspb.2010.0678 PMC2982063 20504812

[72] Lessells C (2008) Neuroendocrine control of life histories: what do we need to know to understand the evolution of phenotypic plasticity? Philos Trans R Soc B Biol Sci 363: 1589–1598 10.1098/rstb.2007.0008 PMC2606726 18048290

[73] Adkins-Regan E (2008) Do hormonal control systems produce evolutionary inertia? Philos Trans R Soc B Biol Sci 363: 1599–1609 10.1098/rstb.2007.0005 PMC2606723 18048293

[74] Martin LB, Brace AJ, Urban A, Coon CAC, Liebl AL (2012) Does immune suppression during stress occur to promote physical performance? J Exp Biol 215: 4097–4103 10.1242/jeb.073049 22933612

[75] Lendvai ÁZ, Chastel O (2008) Experimental mate-removal increases the stress response of female house sparrows: the effects of offspring value? Horm Behav 53: 395–401 10.1016/j.yhbeh.2007.11.011 18191129

[76] Bókony V, Lendvai ÁZ, Liker A, Angelier F, Wingfield JC, et al (2009) Stress Response and the Value of Reproduction: Are Birds Prudent Parents? Am Nat 173: 589–598 10.1086/597610 19281425

[77] MacDougall-Shackleton SA, Dindia L, Newman AEM, Potvin DA, Stewart KA, et al (2009) Stress, song and survival in sparrows. Biol Lett 5: 746–748 10.1098/rsbl.2009.0382 19605381 PMC2827981

[78] Goutte A, Antoine É, Chastel O (2011) Experimentally delayed hatching triggers a magnified stress response in a long-lived bird. Horm Behav 59: 167–173 10.1016/j.yhbeh.2010.11.004 21087608

[79] Masoro EJ (2005) Overview of caloric restriction and ageing. Mech Ageing Dev 126: 913–922 10.1016/j.mad.2005.03.012 15885745

[80] Koolhaas JM, de Boer SF, Coppens CM, Buwalda B (2010) Neuroendocrinology of coping styles: Towards understanding the biology of individual variation. Front Neuroendocrinol 31: 307–321 10.1016/j.yfrne.2010.04.001 20382177

[81] Spencer KA, Evans NP, Monaghan P (2009) Postnatal stress in birds: a novel model of glucocorticoid programming of the hypothalamic-pituitary-adrenal axis. Endocrinology 150: 1931–1934 10.1210/en.2008-1471 19095740

[82] Conti G, Hansman C, Heckman JJ, Novak MFX, Ruggiero A, et al (2012) Primate evidence on the late health effects of early-life adversity. Proc Natl Acad Sci 109: 8866–8871 10.1073/pnas.1205340109 22615410 PMC3384158

[83] Love OP, McGowan PO, Sheriff MJ (2013) Maternal adversity and ecological stressors in natural populations: the role of stress axis programming in individuals, with implications for populations and communities. Funct Ecol 27: 81–92 10.1111/j.1365-2435.2012.02040.x

[84] Schmidt KL, Macdougall-Shackleton EA, Soma KK, Macdougall-Shackleton SA (2014) Developmental programming of the HPA and HPG axes by early-life stress in male and female song sparrows. Gen Comp Endocrinol 196: 72–80 10.1016/j.ygcen.2013.11.014 24291303

[85] Dingemanse NJ, Both C, Drent PJ, Tinbergen JM (2004) Fitness consequences of avian personalities in a fluctuating environment. Proc R Soc Lond B Biol Sci 271: 847–852 10.1098/rspb.2004.2680 PMC1691663 15255104

[86] Wolf M, Doorn GS van, Weissing FJ (2008) Evolutionary emergence of responsive and unresponsive personalities. Proc Natl Acad Sci 105: 15825–15830 10.1073/pnas.0805473105 18838685 PMC2572984

[87] Chevin L-M, Lande R (2013) Evolution of Discrete Phenotypes from Continuous Norms of Reaction. Am Nat 182: 13–27 10.1086/670613 23778223

[88] Price TD, Qvarnström A, Irwin DE (2003) The role of phenotypic plasticity in driving genetic evolution. Proc R Soc Lond B Biol Sci 270: 1433–1440 10.1098/rspb.2003.2372 PMC1691402 12965006

[89] Nussey DH, Wilson AJ, Brommer JE (2007) The evolutionary ecology of individual phenotypic plasticity in wild populations. J Evol Biol 20: 831–844 10.1111/j.1420-9101.2007.01300.x 17465894

[90] Gomez-Mestre I, Jovani R (2013) A heuristic model on the role of plasticity in adaptive evolution: plasticity increases adaptation, population viability and genetic variation. Proc R Soc B Biol Sci 280. Available: http://rspb.royalsocietypublishing.org/content/280/1771/20131869. Accessed 2013 Sept 26. 10.1098/rspb.2013.1869 PMC3790484 24068357

[91] Brommer JE (2013) On between-individual and residual (co)variances in the study of animal personality: are you willing to take the “individual gambit”? Behav Ecol Sociobiol 67: 1027–1032 10.1007/s00265-013-1527-4

