# Supplementary figures and images for: Experimental Food Restriction Reveals Individual Differences in Corticosterone Reaction Norms with No Oxidative Costs
Source: PLoS One. 2014 Nov 11;9(11):e110564. doi: 10.1371/journal.pone.0110564 (PMC4227652; doi:10.1371/journal.pone.0110564)

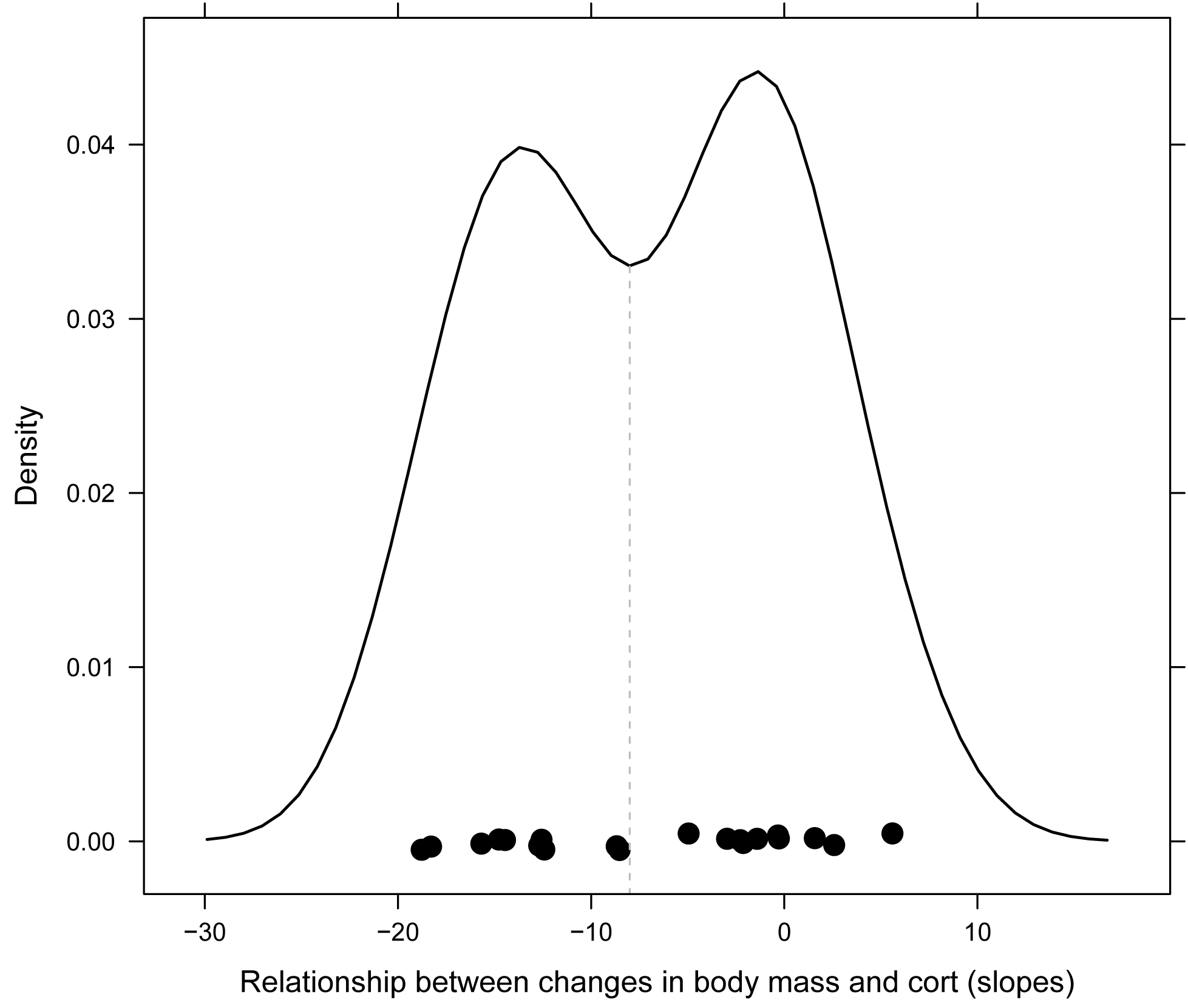

Supplement: Figure S1 — (TIF) [file pone.0110564.s001.tif]
