# Supplementary material for: Experimental Food Restriction Reveals Individual Differences in Corticosterone Reaction Norms with No Oxidative Costs
Source: PLoS One. 2014 Nov 11;9(11):e110564. doi: 10.1371/journal.pone.0110564 (PMC4227652; doi:10.1371/journal.pone.0110564)
Supplement: Table S1 — (DOCX) [file pone.0110564.s002.docx]

**Table S1**. Morphological and physiological measures at capture or at the beginning of the experiment. Parameter estimates and 95% credibility intervals (CI) were generated in MCMCglmm models with treatment on week 1 as a fixed effect, where the control treatment was the reference factor level, therefore the estimates show how birds that started the experiment with the food-restricted diet differed from the birds that started with the control diet.

| id | Week | Estimate | l-95% CI | u-95% CI |
| --- | --- | --- | --- | --- |
| Tarsus (mm) | at capture | 0.43 | -0.40 | 1.29 |
| Body mass (g) | at capture | 0.97 | -0.13 | 2.24 |
| Body mass (g) | beginning of week 1 | 0.7 | -0.52 | 2.06 |
| Corticosterone (ng/ml) | beginning of week 1 | -0.05 | -0.76 | 0.57 |
| Oxidative damage (mg/mL) | beginning of week 1 | -1.92 | -9.77 | 5.48 |
| Total Antioxidant Capacity (mM) | beginning of week 1 | -0.41 | -24.02 | 23.96 |
| Reactive Oxygen Metabolites (mM) | beginning of week 1 | -0.92 | -6.87 | 5.37 |
